# Flame-Retardant and Transparent Unsaturated Polyester Based on P/N Liquid Flame Retardants and Modified Halloysite Nanotubes

**DOI:** 10.3390/ma17030761

**Published:** 2024-02-05

**Authors:** Yanli Dou, Aixun Ju, Zheng Zhong, Yutong Huo, Weiguo Yao

**Affiliations:** The Ministry of Education Key Laboratory of Automotive Material, College of Materials Science and Engineering, Jilin University, Changchun 130025, China; douyl@jlu.edu.cn (Y.D.); juax21@mails.jlu.edu.cn (A.J.); zhongzheng22@mails.jlu.edu.cn (Z.Z.); huoyt22@mails.jlu.edu.cn (Y.H.)

**Keywords:** unsaturated polyester resin, flame retardants, DOPO, halloysite nanotube

## Abstract

Unsaturated polyester resin (UPR) with excellent flame retardant is mainly obtained by adding large amounts of flame retardants, usually at the expense of mechanical properties. In this work, a reactive flame retardant containing phosphorus and nitrogen (DOPO-N) was successfully synthesized and incorporated in UPR as a crosslinker. The mechanical and flame-retardant properties of UPR composites were enhanced. UPR/30DOPO-N passed a UL-94 V-1 rating with a limiting oxygen index (LOI) of 30.8%. The tensile strength of UPR/30DOPO-N increased by 24.4%. On this basis, a small amount of modified HNTs (VHNTs) was added to further improve the flame-retardant properties of the composite. With the introduction of 3 wt% VHNTs, the composite passed the UL-94 V-0 rating. The peak of heat release rate (PHRR) and total heat release (THR) of it decreased by 60.7% and 48.3%, respectively. Moreover, the detailed flame-retarding mechanism of DOPO-N and VHNTs was investigated by thermogravimetric infrared spectroscopy (TG-IR), Raman spectra, and X-ray photoelectron spectroscopy (XPS). It was found that DOPO-N played a role in quenching the flame in the gas phase and cooperated with VHNTs to enhance the barrier effect in the condensed phase.

## 1. Introduction

Unsaturated polyester resin (UPR) is widely used in multiple areas such as chemical engineering, construction, transportation, and aerospace due to its low cost, ease of processing, excellent mechanical properties, chemical corrosion resistance, and weather resistance [1,2,3,4]. However, pure UPR composed of a large number of hydrocarbons is the most flammable among common thermosetting resin materials [5]. Once burned, it releases a large amount of heat and smoke, posing a serious threat to human life, which seriously limits its application in certain fields [6]. For this reason, a number of flame retardants have been prepared to improve the flame retardancy and thermal stability of UPR [7,8,9,10,11,12].

According to whether the added flame retardants react with unsaturated groups, they are divided into blended flame retardants and reactive flame retardants. Various types of blended flame retardants have been prepared, such as organic flame retardants [13,14,15], inorganic flame retardants [16,17], and metal flame retardants [18,19,20]. In order to achieve the desired flame-retardant performance and thermal stability, we can also choose two of them to be used in combination and adjust their addition ratio [17,21,22]. However, when a large number of inorganic flame retardants or metal flame retardants are added to the substrate to achieve a better flame-retardant effect, the interface compatibility between flame retardant and the matrix will become worse because of the viscosity of the system. Then, the mechanical properties of composite materials will deteriorate [23,24,25]. The reactive flame retardants participate in the UPR three-dimensional network structure by crosslinking with the matrix [26]. In this way, the problem of poor compatibility can be avoided, and even the mechanical properties of composite materials will be improved [27]. Most reactive flame retardants for UPR are designed to contain C=C structures with phosphorous or nitrogen [27,28]. Organic phosphorous compounds that are suitable for modification as reactive flame retardants include phosphate esters, ammonium polyphosphate (APP), phosphine oxide, and 9-10-dihydro-9-oxygen-10-phosphate-10-oxide (DOPO) [29,30,31,32,33,34]. Among them, DOPO and its derivatives not only have a high flame retardant effect but also can be easily modified to introduce other elements or functional groups [35,36]. Yuan Cao et al. modified DOPO derivatives and introduced C=C structures to prepare TDCAA-DOPO reactive flame retardants with symmetric structure. The results showed that UPR20 (TDCAA-DOPO content: 20 wt%) achieved an LOI value of 27.2%, and the compatibility between the original phosphorus-containing flame retardant and the matrix was enhanced [37]. Fukai Chu et al. successfully prepared three different polymer flame retardants by modifying DOPO with different monomers (PCH_3_PG, PBPG, and POPG), introducing phosphorus elements and different functional groups to improve flame retardancy and compatibility. The UPR composites were able to reach a V-0 rating of 30% of LOI with 20 wt% of PCH_3_PG, while the mechanical properties of flame retardant composite were decreased [38]. According to previous studies, the onset degradation temperature (T_5%_) of some UPR composite containing phosphorous organic flame retardants was reduced. This was because the P-O structures in composite were unstable and would degrade earlier than the matrix during the heating process [39,40]. Furthermore, the amount of smoke released might increase because of the existence of most organic compounds. Therefore, reactive flame retardants combined with inorganic nanoparticles are a good strategy to improve the thermal stability of composite materials.

Herein, a novel reactive liquid flame retardant containing phosphorus, nitrogen, and C=C structure (DOPO-N) was synthesized by Atherton-Todd reaction, which can be used as a crosslinking point in UPR to improve the mechanical properties and flame retardancy of composite materials. On this basis, the halloysite nanotubes modified by vinyl silane coupling agent (VHNTs) was introduced into the system to reduce the effect of phosphorous flame retardants on the thermal stability of the composites. Due to the combined effect of DOPO-N and VHNTs, the peak of heat release rate of the composite was significantly reduced, and the smoke release was suppressed.

## 2. Experimental

### 2.1. Materials

The unsaturated polyester resin (UPR, type 196) was provided by Jining Huakai Resin Co., Ltd. (Jining, China). The 9,10-dihydro-9-oxa-10-phosphaphenanthrene-10-oxide (DOPO), carbon tetrachloride (CCl_4_), and vinyltrimethoxysilane (VTMS) were procured from Shanghai Maclin Biochemical Technology Co., Ltd. (Shanghai, China). N-(2-Hydroxyethyl)acrylamide (HEAA), triethylamine (Et_3_N), and benzoyl peroxide were purchased from Shanghai Aladdin Biochemical Technology Co., Ltd. (Shanghai, China). HNTs were procured from Guangzhou Runwo Material Technology Co., Ltd. (Guangzhou, China). Dichloromethane (DCM), ethanol, sodium sulfate anhydrous (Na_2_SO_4_), and sodium chloride (NaCl) were provided by Tianjin Xinbote Chemical Co., Ltd. (Tianjin, China).

### 2.2. Synthesis of DOPO-N

**Scheme 1:**
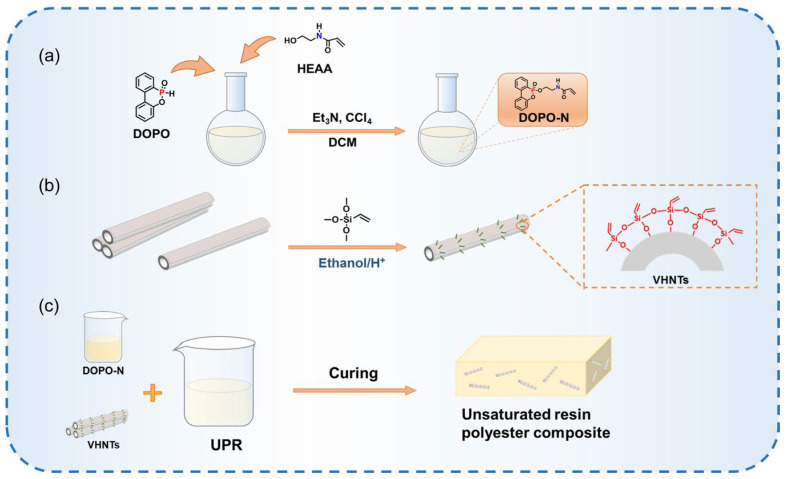
Schematic diagram of the preparation process of (**a**) DOPO-N, (**b**) VHNTs, and (**c**) UPR composites.

The synthesis reaction of DOPO-N is shown in Scheme 1a. DCM (50 mL), DOPO (10 mmol), and Et_3_N (11 mmol) were added in that order while being stirred into a flask. HEAA (11 mmol) was added to the mixture after the DOPO completely dissolved, and then the mixture was cooled to 0 °C in an ice bath. After that, CCl_4_ (11 mmol) was added drop by drop to the mixture and reacted at room temperature for 12 h. The mixture was diluted with DCM and extracted with distilled water and saturated salt water. The organic layer was dried with Na_2_SO_4_ and filtered. After the solvent was removed, the yellow liquid DOPO-N was obtained.

### 2.3. Preparation of VHNTs

The preparation of VHNTs is shown in Scheme 1b. Silanized halloysite nanotubes (VHNTs) were obtained by silanizing pure HNTs. Briefly, ethanol (30 mL), deionized water (3 mL), and acetic acid (0.06 mL) were poured into a beaker to keep the pH of the solution at 4.5–5.5. Then, 2 g HNTs were added to the solution, and 0.5 g VTMS was added dropwise after the HNTs were dispersed evenly. The mixture was magnetically stirred at 50 °C for 5 h. The product obtained was washed to neutrality with deionized water and dried in an oven at 80 °C overnight. It was ground into powder with a mortar and pestle before use.

### 2.4. Preparation of UPR Composites

The formulations of UPR composites are shown in Table 1. Firstly, pure UPR was mixed with a quantitative amount of the flame retardant under stirring at 75 °C. Then, 2% BPO was added into the system, and stirring was continued until the hardener was completely dissolved; then, we poured the mixture into the mold in an oven at 80 °C for 1 h, 100 °C for 2 h, and 120 °C for 1 h. The composite resin was completely cooled and demolded for subsequent testing.

### 2.5. Characterization

The structural composition of the flame retardants was characterized by a Fourier transform infrared spectrometer (FTIR, TENSOR27, Karlsruhe, Germany) using KBr tablets. The wave number ranged from 4000 to 500 cm^−1^. The times of scans were 64.

^1^H or ^31^P nuclear magnetic resonance (NMR, Bruker, Saarbruecken, Germany) experiments were performed by a Bruker AVANCE NEO-600M spectrometer, choosing the deuterated chloroform as a solvent.

The thermal stability of UPR and UPR composites under N_2_ and air atmospheres was analyzed with a thermogravimetric analyzer (TGA, HTG-1, Beijing, China). The gas flow rate was 50 mL/min. The experimental process was heated from 40 °C to 800 °C with a heating speed of 10 °C/min. The purity of N_2_ ≥ 99.999%.

The mechanical properties of UPR and UPR composites were tested with a universal testing machine (WSM-5KN electronic universal testing machine, Beijing, China), and the displacement velocity was 2 mm/min according to GB/T 2567-2008 [41]. The samples were dumbbell tensile test splines with a standard distance of 25 mm, and each group of samples was measured at least 10 times.

According to the ASTMD2863-97 standard [42], the limiting oxygen index values (LOI) of UPR and UPR composites were tested with the JF-3 limiting oxygen index meter with sample sizes of 120 mm × 6.5 mm × 3.2 mm. LOI calculation formula: LOI = [O_2_]/([O_2_] + [N_2_]).

According to the ASTMD3801 standard [43], the vertical combustion performance of the UPR and UPR composites was obtained by a vertical combustion tester (UL-94, China), and the sample sizes were 125 mm ×12.7 mm × 3.2 mm and 125 mm ×12.7 mm × 1.0 mm.

The combustion behavior of UPR and UPR composites was measured with a conical calorimeter test (CCT). Standard sample size was 100 mm × 100 mm × 3 mm.

The gas phase products of the UPR and UPR composites in the pyrolysis process were analyzed in a N_2_ atmosphere by TG-FTIR (TG, 209F3; FTIR, TENSOR27, iCone, London, UK) from room temperature to 800 °C with a heating rate of 15 °C/min.

The morphologies of cross-section and carbon residues were observed by a field emission scanning electron microscope (SEM, HITACHI TM4000Plus, Tokyo, Japan).

The carbonization degree of char residue of UPR composites was evaluated by Raman spectroscopy (Thermo Scientific DXR3Xi, Waltham, MA, USA).

The X-ray photoelectron spectroscopy (XPS, Waltham, MA, USA) test used the Thermo Scientific K-Alpha to test the X-ray photoelectron spectroscopy of char residue.

## 3. Results and Discussion

### 3.1. Characterizations of DOPO-N and VHNTs

**Figure 1:**
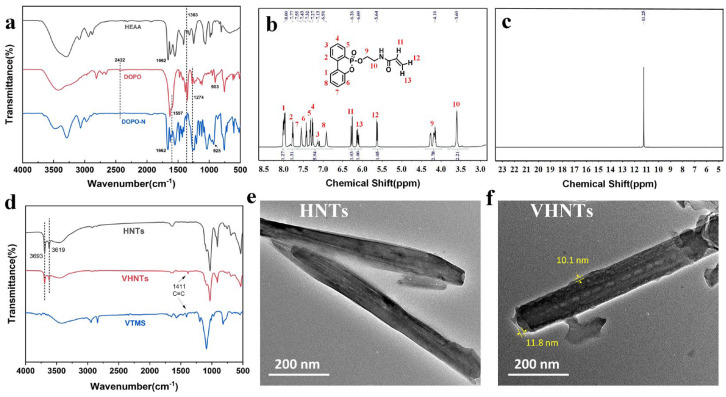
FTIR spectra of (**a**) DOPO-N and (**d**) VHNTs; (**b**) ^1^H NMR and (**c**) ^31^P NMR spectra of DOPO-N; TEM images of (**e**) HNTs and (**f**) VHNTs.

DOPO-N was prepared by the Atherton–Todd reaction. In order to explain its structure in more detail, FTIR spectroscopy was used to analyze DOPO HEAA and DOPO-N. The results are shown in Figure 1a [32,44]. For HEAA, the absorption peak at 1660 cm^−1^ was ascribed to the stretching vibration of C=O; the wide absorption peaks from 3000 cm^−1^ to 3500 cm^−1^ were attributed to -OH [45]. For DOPO, the characteristic absorption peaks at 2432 cm^−1^, 1597 cm^−1^, and 1274 cm^−1^ belong to P-H, P-Ph, and P-O, respectively [31,46,47]. It was found that the characteristic peaks of DOPO and HEAA were shown in the FTIR spectra of DOPO-N, while the absorption peak of P-H disappeared [48]. This suggested that the P-H bond was consumed in the Atherton–Todd reaction.

To confirm the successful preparation of DOPO-N, it was performed by ^1^H and ^31^P NMR spectra. As shown in Figure 1b,c, the aromatic hydrogen protons of DOPO appeared at 6.91–8.00 ppm [38]. There were three peaks at 6.26 ppm, 6.09 ppm, and 5.64 ppm corresponding to H in -CH=CH_2_ from HEAA [49,50]. The peaks at 4.16 ppm and 3.60 ppm were attributed to H in the CH_2_-CH_2_ structure. Meanwhile, the ^31^P NMR spectra of DOPO-N showed a clean single peak at 11.25 ppm regarding the phosphorus atom, indicating the successful preparation of DOPO-N.

VHNT modified particles containing C=C structure were synthesized through a series of hydrolysis and co-condensation reactions among the VTMS and HNTs, as illustrated in Scheme 1 [51]. The FTIR spectra of HNTs, VTMS, and VHNTs are shown in Figure 1d. The peaks located at 3693 cm^−1^ and 3619 cm^−1^ were considered to be the stretching vibrations of internal and external Al-OH, respectively [52,53]. In term of VHNTs, a new vibration band appeared at 1411 cm^−1^, which belonged to the in-plane bending vibration absorption peak of C=C, which demonstrated that the modification of HNTs was successful [54].

The TEM images of HNTs exhibited a nanotube structure with a smooth exterior surface, as shown in Figure 1e. For VHNTs, a rough organic layer was polymerized on the outer surface of HNTs by VTNS without deterioration of the tubular structure of HNTs. And the thickness of the organic layer reached up to near 11.8 nm.

### 3.2. Thermal Stability of UPR and Its Composites

**Figure 2:**
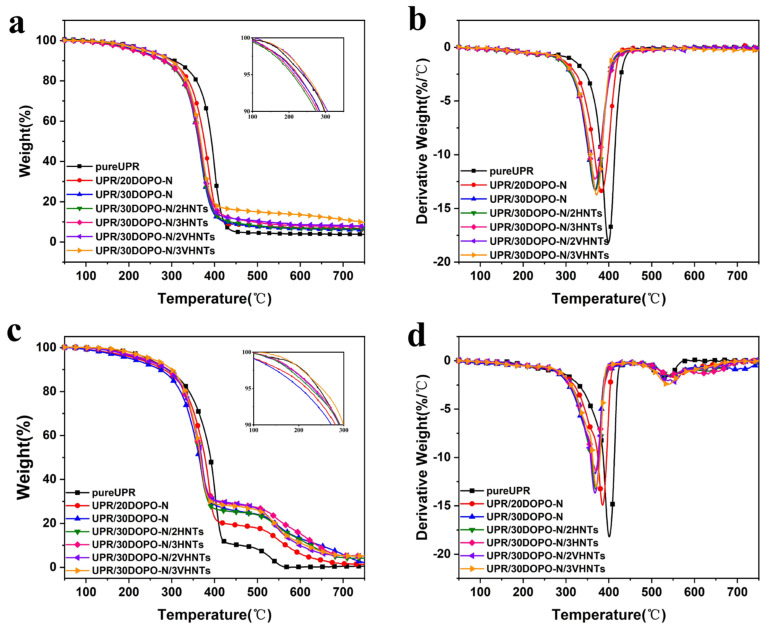
TGA and DTG curves of pure UPR and UPR composites in N_2_ (**a**,**b**) and air (**c**,**d**).

The thermal stability of UPR and UPR composites was investigated by TGA, and the results are shown in Figure 2 and Table S2. In general, the onset degradation temperature was defined as the temperature with a 5 wt% of weight loss and denoted as T_5%_. The temperature at the maximum mass loss rate was denoted as T_max_. The degradation processes of all samples in the N_2_ atmosphere presented only one stage, attributed to the scission of unsaturated polyester and polystyrene chain segments, with a strong DTG peak at 350–400 °C [29]. For UPR, its T_5%_ and T_max_ in N_2_ were 240 °C and 400 °C, respectively, and the residue mass percentage at 750 °C was 3.26%. UPR/20DOPO-N and UPR/30DOPO-N had lower T_5%_ and T_max_ than pure UPR. The T_5%_ of UPR/20DOPO-N and UPR/30DOPO-N was reduced attributing to the early thermal decomposition of DOPO-N [55,56,57]. Compared to pure UPR (3.26%), the residue of UPR/20DOPO-N and UPR/30DOPO-N at 750 °C increased to 5.95% and 6.04%, respectively, indicating that DOPO-N had played a role in promoting the char formation. Furthermore, the T_5%_ of UPR/30DOPO-N/2VHNTs and UPR/30DOPO-N/3VHNTs were increased compared to other samples, including pure UPR. And the residue mass percentage at 750 °C in N_2_ of UPR/30DOPO-N/2VHNTs and UPR/30DOPO-N/3VHNTs gradually enhanced to 7.78% and 9.67%, respectively. We found that UPR/30DOPO-N/VHNTs exhibited better thermal stability and higher carbon residue than UPR/30DOPO-N/HNTs when the same number of HNTs or VHNTs were added to UPR/30DOPO-N systems. This was due to the good dispersion and compatibility of the VHNTs in the UPR matrix.

In air atmosphere, new secondary degradation phenomena appeared for UPR and UPR composites in the higher temperature range (480–650 °C), which were ascribed to the further thermo-oxidative degradation of the primary products formed at the first stage (250–450 °C) [13]. The decrease in T_5%_ of the composites compared with pure UPR in air was due to the decomposition of DOPO-N, which was basically consistent with the mechanism under N_2_ atmosphere. Flame-retardant UPR composites still possessed higher carbon residue. The residue of UPR/30DOPO-N at 750 °C increased from 0.52% (pure UPR) to 2.18%, and the residue of UPR/30DOPO-N/3VHNTs at 750 °C increased from 2.18% (UPR/30DOPO-N) to 5.24%. As a consequence, the introduction of DOPO-N and VHNTs promoted the formation of more stable char layers, which inhibited the oxygen and heat transfer, thus suppressing the matrix from the thermal/thermo-oxidative decomposition.

### 3.3. Mechanical Properties of UPR Composites

**Figure 3:**
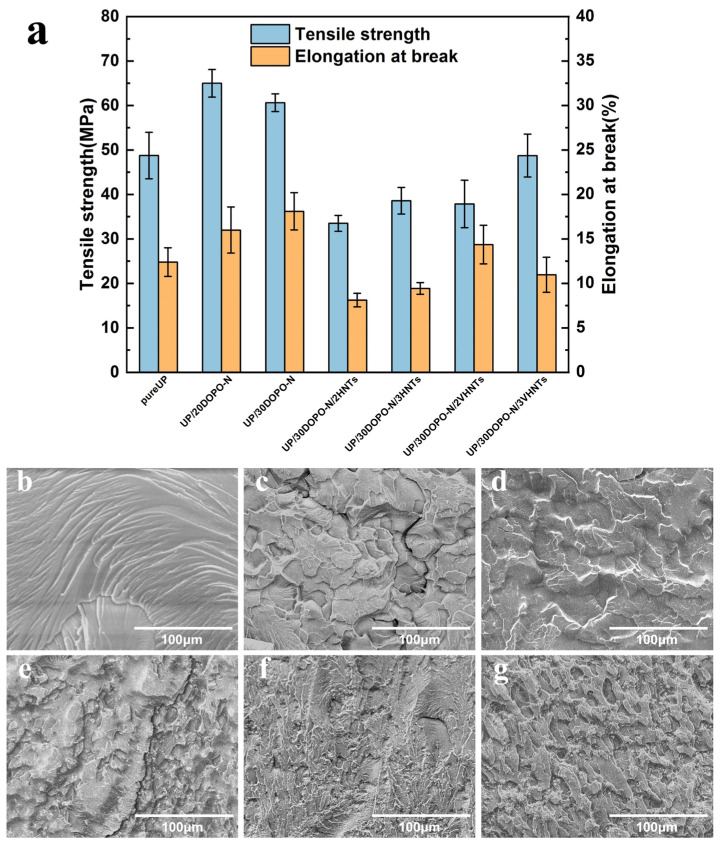
(**a**) Tensile strength and elongation at break of UPR composites. The SEM images of the sections of (**b**) UPR, (**c**) UPR/20DOPO-N, (**d**) UPR/30DOPO-N, (**e**) UPR/30DOPO-N/3HNTs, (**f**) UPR/30DOPO-N/2VHNTs, and (**g**) UPR/30DOPO-N/3VHNTs after tensile tests.

**Figure 4:**
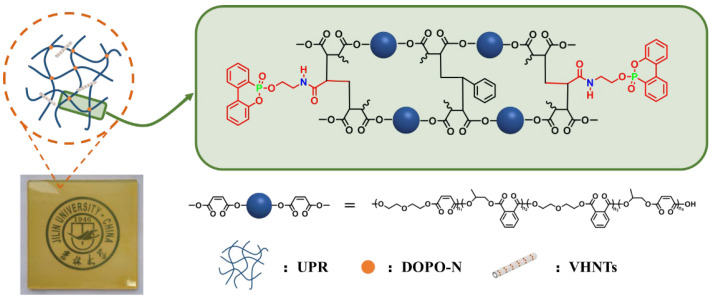
The possible crosslinking pattern of UPR composites.

The mechanical properties of UPR composites are shown in Figure 3a, while the fracture morphologies of these composites are shown in Figure 3b–g. As shown in Figure 3, the tensile strength of the pure UPR was 48.74 MPa. With the introduction of liquid DOPO-N, the tensile strength and elongation at the break of the composites were both increased significantly, and the fracture surface became rougher, as shown in Figure 3b,c. The tensile strength of the UPR/20DOPO-N and UPR/30DOPO-N were 65.01 MPa and 60.61 MPa, respectively. Basically, DOPO-N containing a C=C structure has good compatibility with UPR. Besides that, the double bond of DOPO-N was able to participate in the cross-linking reaction of the UPR matrix, which introduced a rigid structure of biphenyl into the molecular chain of UPR and increased the crosslinking degree; as a result, the mechanical properties were improved. The possible crosslinking reaction of DOPO-N and UPR was shown in Figure 4 [58]. The addition of inorganic particles usually leads to a decline in mechanical properties of composite materials due to issues of compatibility and cluster [59]. The tensile strength of the composite UPR/30DOPO-N/3HNTs was decreased to 38.58 MPa. Introduction of a double-bond structure on the HNT surface can improve the compatibility between HNTs and UPR, as well as the dispersion state in UPR [60]. The tensile strength of the composite with 3 wt% modified HNTs was increased by 26.3%, compared to UPR/30DOPO-N/3HNTs. In addition, all samples had good light transmission, as shown in Figure S1.

### 3.4. Flame Retardancy of UPR Composites

In order to evaluate the flame retardancy of composites, limiting oxygen index (LOI) and UL-94 vertical flammability were generally selected as reliability parameters, as shown in Table 2 and Figure S2. The thickness of the samples was 3.2 mm. Pure UPR burned quickly and violently. After adding DOPO-N, the flame of the composite with 20 wt% DOPO-N weakened, but the burning time was long. UPR/20DOPO-N was assessed as no rating (NR). In contrast, the composite with the addition of 30 wt% DOPO-N exhibited self-extinguishing performance at 24 s after being ignited the second time, without any drops, reaching the UL-94 V-1 grade. On this basis, after a certain amount of HNTs or VHNTs, it was found that the UPR composites reached UL-94 V-0 grade, and the self-extinguishing performance was further enhanced. The LOI of UPR and UPR composites were measured as shown in Table 2. The LOI of pure UPR material was only 23.0%, which was flammable material. When the content of DOPO-N reached 20% and 30%, the LOI of the UPR/20DOPO-N and UPR/30DOPO-N reached 29.0% and 30.8%, respectively. The LOI of UPR/30DOPO-N/2VHNTs and UPR/30DOPO-N/3VHNTs reached 31.1% and 31.3% after the further addition of inorganic flame retardant. The above results indicated that the introduction of DOPO-N and VHNTs contributed to reducing the self-extinguishing time and increasing the LOI values of composites, thereby improving the fire-safety performance of UPR.

According to ASTMD3801 standard, we also prepared samples with sizes of 125 mm × 12.7 mm × 1.0 mm for the UL-94 test. However, those samples did not pass the UL-94 test under the same standards and conditions.

### 3.5. Combustion Behavior of UPR Composites

**Figure 5:**
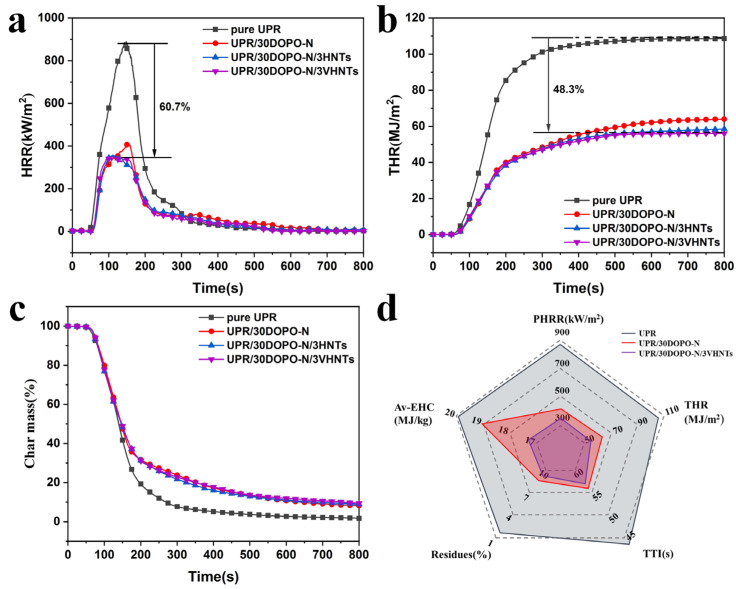
CCT curves of UPR and UPR composites: (**a**) HRR, (**b**) THR, and (**c**) mass. (**d**) Spider web plot of comparison with important flame-retardant parameters for UPR, UPR/30DOPO-N, and UPR/30DOPO-N/3VHNTs.

The combustion performance of the composites was evaluated by the cone calorimeter test (CCT). The main parameters were peak heat release rate (PHRR), total heat release rate (THR), and average effective heat of combustion (Av-EHC) [61,62]. As shown in Figure 5 and Table S2, the ignition time (TTI) of the UPR was 44 s, which increased in the flame retardant UPR composites. This indicated that the introduction of DOPO-N and HNTs improved the fire resistance performance of UPR [63,64].

As shown in Figure 5a,b, the pure UPR was highly flammable with a high PHRR (890.28 kW/m^2^) and THR (108.92 MJ/m^2^). With the combined effect of DOPO-N and HNTs, the PHRR and THR of the flame retardant UPR composites were reduced significantly. The PHRR and THR of UPR/30DOPO-N/3VHNTs decreased to 350.11 kW/m^2^ and 56.21 MJ/m^2^, decreasing by 60.7% and 48.3%, respectively.

As shown in Table S2, the average efficient heat of combustion (Av-EHC) decreased from 20.07 MK/kg for pure UPR to 17.32 MK/kg for UPR/30DOPO-N/3VHNTs, due to the production of non-flammable gases in the gas phase and incomplete combustion of the gas phase volatiles [65,66]. On the one hand, the DOPO-N in UPR composites would release volatile phosphide, NH_3_, NO_2_, and other non-flammable gases during the combustion. On the other hand, when DOPO groups decomposed, they released phosphate-containing free radicals to interrupt the radical chain reaction, resulting in incomplete combustion of the volatiles in the gas phase [67,68,69]. The carbon residue rate of UPR/30DOPO-N/3VHNTs increased from 1.69% to 9.49%. Furthermore, Figure 5d showed a comparison of important flame-retardant parameters of UPR, UPR/30DOPO-N, and UPR/30DOPO-V/3VHNTs.

### 3.6. Flame Retardant Mechanism

**Figure 6:**
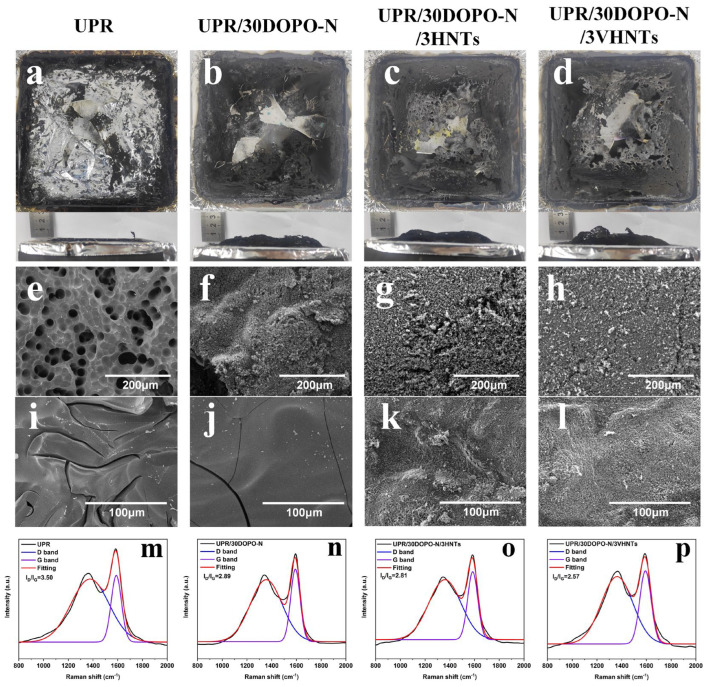
(**a**–**d**) Photographs and SEM images of the (**e**–**h**) external surface and (**i**–**l**) internal surface of the residue char after the cone calorimeter test. Raman spectra of the residue char after CCT (**m**–**p**).

The structure and morphology of carbon slag were studied to understand the flame retardancy mechanism of DOPO-N and HNTs. The outside and inside morphologies of residual char after CCT are shown in Figure 6. For pure UPR, there were circular pores distributed on the external surface of the residual char and relatively dense cracks on the internal surface (Figure 6e,i). The thin char layer with cracks was unable to prevent the transfer of heat and the release of volatile products [70,71]. When 30 wt% DOPO-N was incorporated into UPR, the pores no longer appeared on the external surface, instead of a tight and dense char layer; meanwhile, the internal cracks were less than that of pure UPR (Figure 6f,j). It is worth noting that the external char showed remarkable changes when 3 wt% HNTs or VHNTs were added into the UPR/30DOPO-N (Figure 6g,h). The char layer of the UPR/30DOPO-N/3VHNTs became denser, smoother, and with less cracks. At the same time, HNTs or VHNTs can be observed to be tightly packed on the inner surface, acting as a barrier layer to prevent the composites from the erosion of the oxygen and heat (Figure 6k,l) [31,72,73,74].

Raman spectroscopy was used to further evaluate the graphitization degree of the carbonization of pure UPR and UPR/30DOPO-N/3VHNTs after CCT. It was noticeable that there were two peaks in the given spectra, corresponding to the D band and the G band [75,76]. The D peaks represent disordered graphite or glassy carbon, and the G peaks represent an organized and ordered graphitic structure, in which the ratio of the area under the D band to the area under the G band (I_D_/I_G_) is used to calculate the degree of graphitization of the carbon residue; the larger the value of I_D_/I_G_, the higher the degree of disorder of the carbon layer [77,78]. As shown in Figure 6m–p, the I_D_/I_G_ values of pure UPR, UPR/30DOPO-N, UPR/30DOPO-N/3HNTs, and UPR/30DOPO-N/3VHNTs were 3.50, 2.89, 2.81, and 2.57, respectively. The results showed that the char microstructure was improved by the incorporation of DOPO-N and VHNTs.

**Figure 7:**
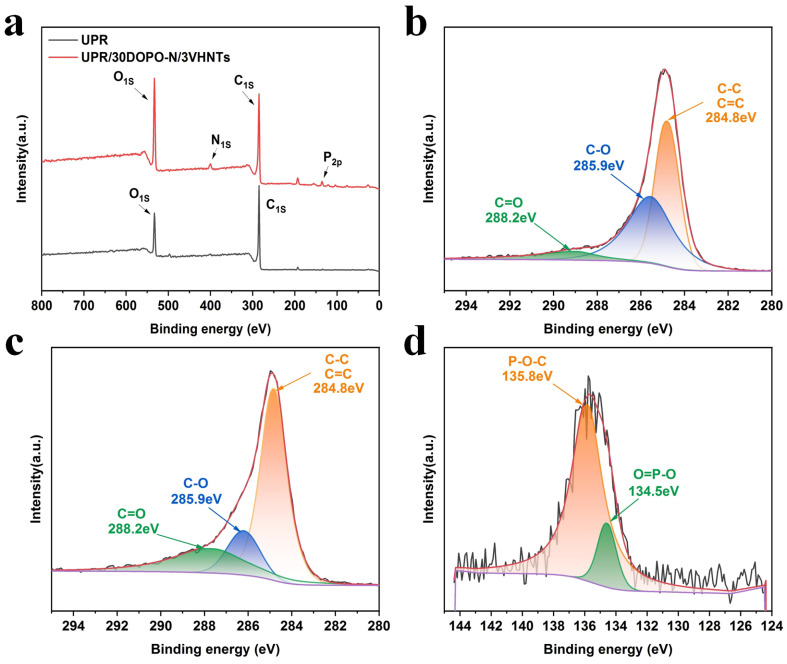
(**a**) Full XPS measurement scan; (**b**) C_1s_ spectra of UPR carbon residue; (**c**) C_1s_ and (**d**) P_2p_ spectra for the UPR/30DOPO-N/3VHNTs.

The analysis of the chemical state of different elements in the residual carbon layer of UPR and UPR/30DOPO-N/3VHNTs composites were performed by the XPS analysis, as shown in Figure 7. There were only carbon (C) and oxygen (O) elements in the residual carbon of UPR, while nitrogen (N) and phosphorus (P) elements were detected in UPR/30DOPO-N/3VHNTs besides the C and O elements. The binding energy of C_1s_ in UPR and UPR/30DOPO-N/3VHNTs residues at 288.2 eV, 285.9 eV, and 284.8 eV belonged to the C=O, C-O, and C=C/C-C, respectively [79]. We found that the C=C/C-C peak in the residues of UPR/30DOPO-N/3VHNTs was stronger than that of UPR, and the integrated area was larger, indicating that more residual carbon layers were formed [80]. For the P_2p_ of UPR/30DOPO-N/3VHNTs residues, the binding energies at 135.8 eV and 134.5 eV were assigned to P-O-C and O=P-O, respectively [81]. It could be demonstrated that phosphoric acid, phosphate ester, and pyrophosphoric acid were produced during combustion and contributed to the catalytic formation of carbon layers.

**Figure 8:**
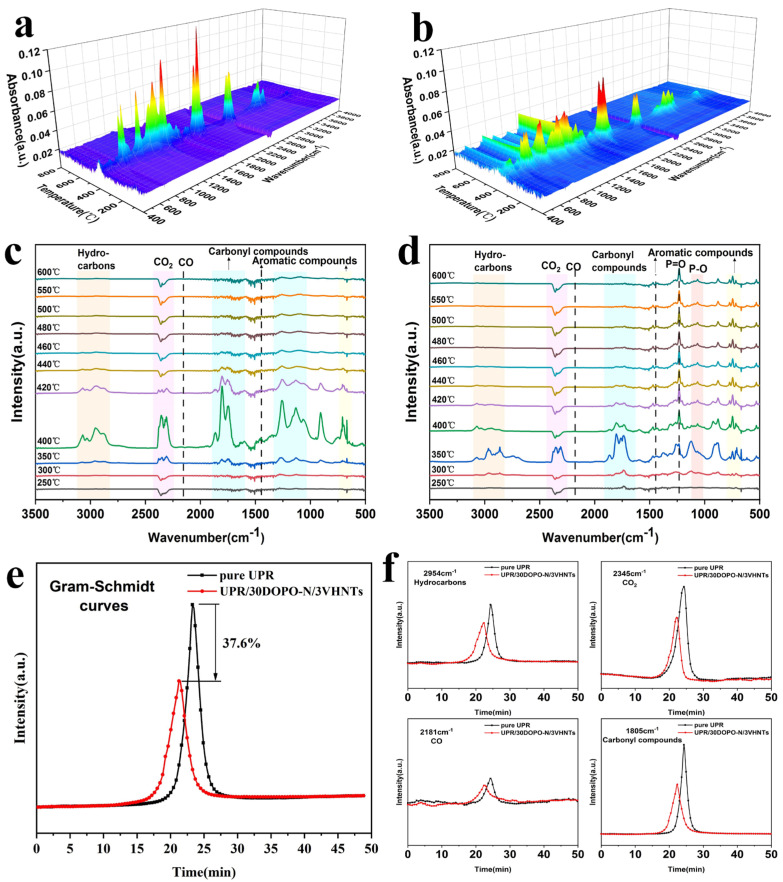
FTIR 3D spectra of (**a**) UPR and (**b**) UPR/30DOPO-N/3VHNTs. FTIR spectra of the decomposition products at different temperatures for (**c**) UPR and (**d**) UPR/30DOPO-N/3VHNTs. Intensity of (**e**) total pyrolysis products and (**f**) hydrocarbons, CO_2_, CO, and carbonyl compounds.

To study the effects of DOPO-N and HNTs on the flame retardants of UPR in the vapor phase, TG-FTIR was employed to analyze the volatile components. As the 3D TG-FTIR in Figure 8 shows, the intensity of each characteristic absorption peak was significantly reduced, and new peaks appeared after incorporating 30 wt% DOPO-N and 3 wt% VHNTs in UPR. The characteristic peaks of UPR and UPR/30DOPO-N/3VHNTs are shown in Figure 8c,d. Gas-phase products of UPR were mainly released at around 400 °C, including CO_2_ (2345 cm^−1^), hydrocarbons (2850–3100 cm^−1^), aromatic compounds (689–833 cm^−1^), and carbonyl compounds (1805 cm^−1^) [82,83,84]. Compared with UPR, the same pyrolysis products of UPR/30DOPO-N/3VHNTs appeared at 350 °C, and the phosphorus-containing compounds (P=O, 1243 cm^−1^; P-O, 1060 cm^−1^) were also detected [85]. Besides that, the total pyrolysis products in UPR/30DOPO-N/3VHNTs decreased by 37.6% compared with UPR, and the intensities of CO_2_ and carbonyl compounds decreased most obviously, as shown in Figure 8e,f. Therefore, the composite UPR/30DOPO-N/3VHNTs had two ways to reduce the vapor phase products. On the one hand, the free radicals generated in the chain reaction were captured by PO_2_• and PO• radicals from the earlier thermal decomposition of DOPO-N [14], thereby achieving gas-phase flame retardancy. On the other hand, the uniformly dispersed VHNTs also formed a physical barrier to protect the matrix during combustion, thus reducing the release of pyrolysis products [72].

## 4. Conclusions

In this work, a novel liquid flame retardant containing phosphorus and nitrogen, DOPO-N, was synthesized and used as a crosslinker in the UPR crosslinking reaction. When 30 wt% DOPO-N was added, the tensile strength of UPR composite increased by 24.4%. UPR/30DOPO-N showed an impressive LOI value of 30.8%. In addition, with the HNTs modified by VTMS, double bonds were introduced on its surface to improve its dispersion in the UPR. With 3 wt% of VHNTs incorporated into UPR/30DOPO-N, PHRR and THR of the composite were reduced by 60.7% and 48.3%, respectively, compared to pure UPR. The LOI of UPR/30DOPO-N/3VHNTs reached 31.3%. The simultaneous addition of DOPO-N and HNTs showed a combined effect on the gas phase and condensate phase, which achieved the purpose of improving the flame retardancy of UPR composites without sacrificing the mechanical properties.

## Figures and Tables

**Table 1:** Formulation of flame retardant UPR composites.

Samples	UPR (g)	DOPO-N (g)	HNTs (g)	VHNTs (g)	BPO (g)
UPR	100	0	0	0	2.00
UPR/20DOPO-N	80	20	0	0	1.60
UPR/30DOPO-N	70	30	0	0	1.40
UPR/30DOPO-N/2HNTs	68	30	2	0	1.36
UPR/30DOPO-N/3HNTs	67	30	3	0	1.34
UPR/30DOPO-N/2VHNTs	68	30	0	2	1.36
UPR/30DOPO-N/3VHNTs	67	30	0	3	1.34

**Table 2:** Data of LOI and UL-94 vertical burning tests of the samples.

Samples	LOI (%)	UL-94		
		Rating	t_1_ + t_2_ (s)	Dripping
UPR	23.0 ± 0.2	NR	50	-
UPR/20DOPO-N	29.0 ± 0.2	NR	50	-
UPR/30DOPO-N	30.8 ± 0.2	V-1	25	-
UPR/30DOPO-N/2HNTs	31.0 ± 0.2	V-0	3	-
UPR/30DOPO-N/3HNTs	31.1 ± 0.2	V-0	2	-
UPR/30DOPO-N/2VHNTs	31.1 ± 0.2	V-0	1	-
UPR/30DOPO-N/3VHNTs	31.3 ± 0.2	V-0	1	-

## Data Availability

Data are contained within the article and supplementary materials.

## Supplementary Materials

The following supporting information can be downloaded at: https://www.mdpi.com/article/10.3390/ma17030761/s1. Table S1. Relevant data of UPR composites in TG test. Table S2. CCT data of UPR and UPR composites. Table S3. Raman test data of carbon residue. Figure S1. Photo of light transmittance of sample. Figure S2. Burning process during UL-94 vertical burning test at different times.
