# Supplementary material for: Flame-Retardant and Transparent Unsaturated Polyester Based on P/N Liquid Flame Retardants and Modified Halloysite Nanotubes
Source: Materials (Basel). 2024 Feb 5;17(3):761. doi: 10.3390/ma17030761 (PMC10856195; doi:10.3390/ma17030761)
Supplement: Supplementary file 1 [file materials-17-00761-s001.zip › materials-2831986-supplementary.pdf]

Table S1. Relevant data of UPR composites in the TG test.

Samples	$T_{5\%}$ (℃)		T_{max} (℃)		Residue (%)-750	
	N_2	Air	N_2	Air	N_2	Air
UPR	240	243	400	401	3.26	0.52
UPR/20DOPO-N	238	218	384	385	5.95	1.42
UPR/30DOPO-N	221	205	369	365	6.04	2.18
UPR/30DOPO-N/2HNTs	218	228	371	368	6.52	4.19
UPR/30DOPO-N/3HNTs	220	235	370	370	7.68	5.11
UPR/30DOPO-N/2VHNTs	244	231	372	368	7.78	5.09
UPR/30DOPO-N/3VHNTs	245	249	372	371	9.67	5.24

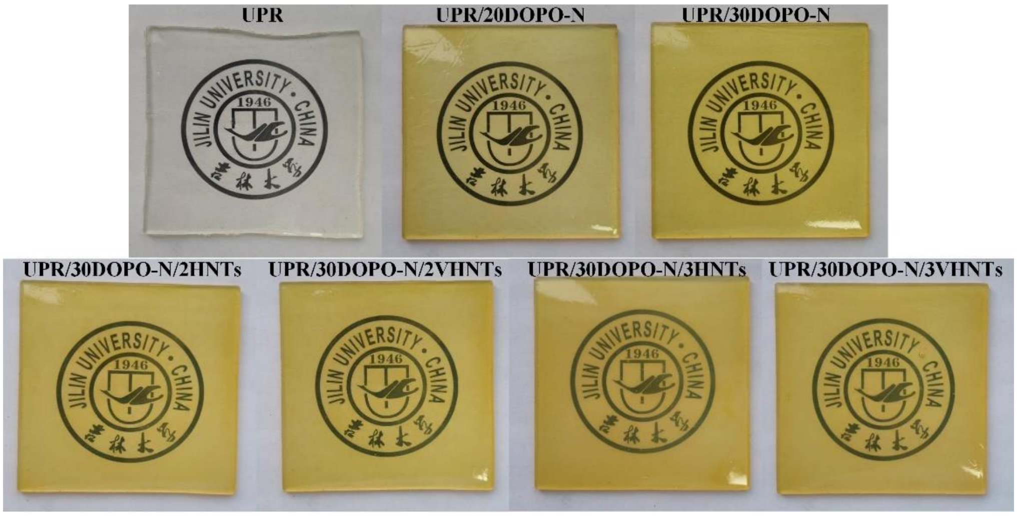

Figure S1. Photo of light transmittance of the sample.

Figure S1 shows the transparency of pure UPR and UPR composites at a thickness of 3mm. It can be seen from the figure that all the patterns were clearly seen through the sample, indicating that all the composites exhibited good transparency in the visible light region. This transparency was due to the good compatibility between the flame retardant and the substrate.

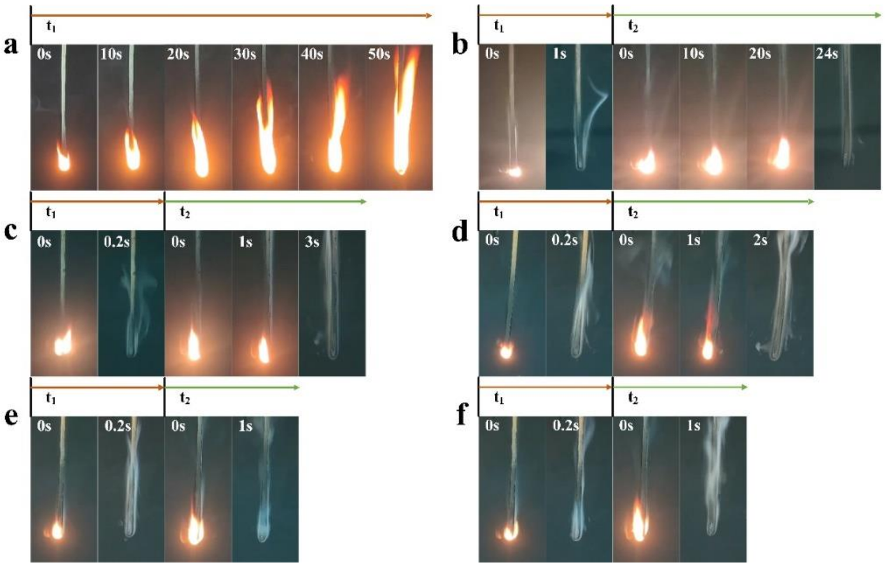

Figure S2. Burning process during the UL-94 vertical burning test at different times: (a) UPR, (b) UPR/30DOPO-N, (c) UPR/30DOPO-N/2HNTs, (d) UPR/30DOPON/3HNTs, (e) UPR/30DOPO-N/2VHNTs, (f) UPR/30DOPO-N/3VHNTs.

Table S2. CCT data of UPR and UPR composites.

Samples	TTI(s)	PHRR (kW/m^2)	THR (MJ/m^2)	Av-EHC (MJ/kg)	Residues (%)
UPR	44	890.28	108.92	20.07	1.69
UPR/30DOPO-N	56	414.17	64.32	19.16	8.19
UPR/30DOPO-N/3HNTs	56	358.10	59.49	19.37	8.83
UPR/30DOPO-N/3VHNTs	57	350.11	56.21	17.32	9.49

TTI: time to ignition; PHRR: peak of heat release rate; THR: total heat release; Av-EHC: average effective heat of combustion; TSP: total smoke production; TSR: total smoke release; Av-COY: average carbon monoxide yield; Av-CO_2Y: average carbon dioxide yield.

Table S3. Raman test data of carbon residue.

Samples	AD($\times10^5$)	AG($\times10^5$)	I_D/I_G	FWHM(D)	FWHM(G)
UPR	26.44	7.56	3.50	362.83	98.16
UPR/30DOPO-N	18.38	6.37	2.89	303.28	89.81
UPR/30DOPO-N/3HNTs	18.09	6.43	2.81	308.37	96.76
UPR/30DOPO-N/3VHNTs	27.13	10.56	2.57	301.81	108.23

AD: the integral area of D peak; AG: the integral area of G peak; I_D/I_G: the ratio of the area under the D bond to the area under the G bond, which is the degree of graphitization; FWHE: full width half maximum.
